# Gold Shell-Isolated Nanoparticles Differentially Affect Neutrophil Antimicrobial Function

**DOI:** 10.1021/acsomega.6c02936

**Published:** 2026-09-29

**Authors:** João Guilherme Beloto Moraes, Ronaldo Silva Alves Júnior, Mirella Boaro Kobal, André Satoshi Ferreira, Sabrina Alessio Camacho, Pedro Henrique Benites Aoki, Karina Alves Toledo

**Affiliations:** † School of Sciences, Humanities and Languages, São Paulo State University (UNESP), Assis, São Paulo 19806-900, Brazil; ‡ Institute of Biosciences, Humanities and Exact Sciences, São Paulo State University (UNESP), São José do Rio Preto 15054-000, Brazil

## Abstract

Gold-based nanomaterials are widely explored for biomedical applications, yet their interactions with innate immune cells remain insufficiently defined. Herein, we evaluated the interaction of gold shell-isolated nanoparticles (AuSHINs) with freshly isolated human neutrophils, integrating physicochemical characterization with uptake, viability, and functional assays. AuSHINs were readily internalized within 5 min, and cell-death profiling showed no increase in necrosis across the tested range, whereas apoptotic signaling became evident from 32 μg/mL and was more pronounced at higher exposures. At lower doses (6.4–32 μg/mL), viability was maintained, and neutrophil effector outputs shifted: AuSHINs acted as chemotactic cues in Boyden chamber assays, reduced neutrophil-mediated bacterial clearance and promoted NET formation to levels comparable to PMA. At higher concentrations, NET release decreased in parallel with reduced viability. By mapping these responses across concentration windows, this work frames how gold–silica nanostructures can redirect neutrophil antimicrobial strategies and highlights the need to evaluate immune function alongside standard cytotoxicity end points when designing and benchmarking nanomaterials intended for biological environments.

## Graphical abstract

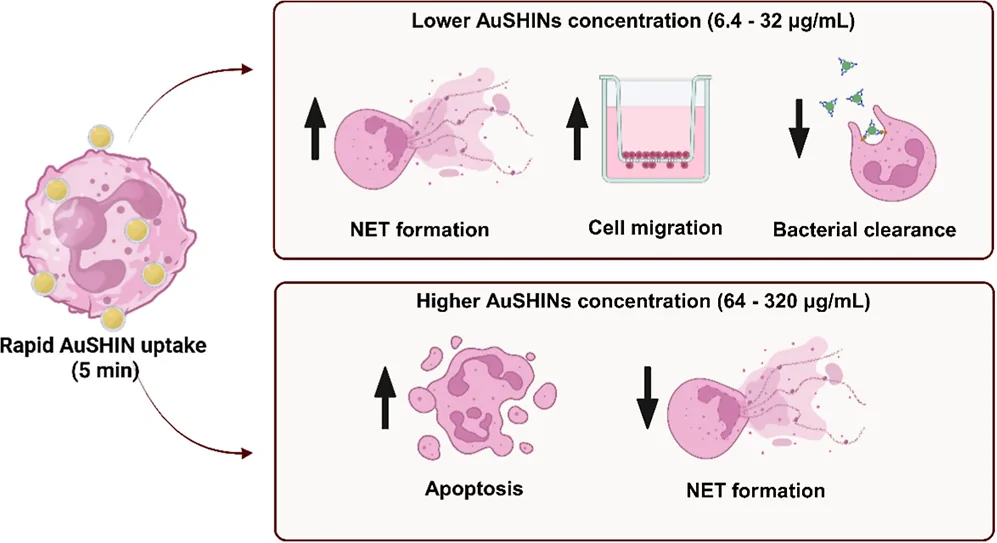

## Introduction

Infectious diseases remain a major global health burden, and the accelerating rise of antimicrobial resistance continues to erode the effectiveness of conventional therapies.^1,2^ This landscape has intensified interest in alternative and adjunct strategies that can either directly constrain pathogens or reshape host defense in a controllable manner. Nanotechnology offers a versatile route toward such interventions because nanoscale materials can be engineered to present defined size, surface chemistry, and interfacial properties that govern interactions with biological systems.

Among nanomaterials, gold nanoparticles (AuNPs) have been widely investigated in biomedicine due to their chemical stability, tunable optical response, and compatibility with surface functionalization. AuNPs can be synthesized through several chemical and biological approaches, including classical citrate reduction, seed-mediated growth, thiol-assisted functionalization, and green synthesis strategies employing plant-derived reducing agents.^3–6^ These preparation methods allow precise control over nanoparticle size, morphology, dispersity, colloidal stability, and surface charge, parameters that critically influence nanoparticle behavior in biological environments.^3,7,8^

In particular, surface functionalization has emerged as a central determinant of nanobio interactions because the outer nanoparticle interface governs interactions with biomolecules, cell membrane, and intracellular pathways.^9^ Function coatings based on thiols, silica, polymers, carbohydrates, antibodies, peptides, and other bioactive molecules have therefore been extensively explored to improve hydrophilicity, colloidal stability, targeting capacity, cellular uptake, and biocompatibility.^3,10^ Importantly, even subtle modifications in surface chemistry or ligand organization can substantially alter nanoparticle internalization, aggregation behavior, intracellular trafficking, and cytotoxicity across different biological systems. Consequently, nanoparticles with similar metallic cores may induce markedly distinct biological outcomes depending on their surface architecture and functionalization strategy.

Gold-based platforms have been explored for antimicrobial and antivirulence effects, including disruption of microbial membranes, inhibition of biofilms, and interference with viral infection processes, as well as for diagnostics and photothermal approaches in localized disease settings.^11–16^ More recently, architectural advances have enabled hybrid designs that combine plasmonic cores with protective shells. Gold shell-isolated nanoparticles (AuSHINs) are particularly attractive because the silica layer can improve colloidal stability and biocompatibility while providing a modular surface for conjugation and environmental control at the nano–bio interface.^17^ However, improved stability does not necessarily translate into immunological neutrality; for translational development, it is essential to define how these constructs engage immune cells under relevant exposure conditions.

Neutrophils represent a primary interface between circulating nanomaterials and innate immunity. As professional phagocytes, they rapidly respond to inflammatory cues and deploy complementary antimicrobial programs, including phagocytosis and the release of neutrophil extracellular traps (NETs).^18–20^ These responses are tightly regulated because neutrophil activation also shapes tissue injury, inflammation resolution, and the balance between intracellular and extracellular microbial control. A growing body of evidence indicates that gold-based nanomaterials can modulate neutrophil behavior, including uptake, phagocytosis, oxidative and degranulation pathways, and NET release.^21^ Yet, reported outcomes vary widely across material designs and experimental conditions, and functional readouts are often interpreted without parallel resolution of cell-death pathways or migratory behavior—parameters that can strongly influence how neutrophils redistribute and operate at sites of nanoparticle exposure.

This question extends beyond antimicrobial contexts. Recent advances in nanomedicine have increasingly explored nanomaterials not only as direct antimicrobial agents but also as regulators of host immune responses, including macrophage polarization and remodeling of inflammatory microenvironment during infectious process.^22^ Gold nanomaterials are also central to photothermal and photodynamic approaches in oncology, where therapeutic benefit depends not only on direct cytotoxic mechanisms but also on how innate immune cells are engaged within the tumor microenvironment.^23–25^ In both infection and cancer, therefore, understanding how gold–silica nanostructures interface with neutrophils is important for anticipating downstream inflammatory consequences and for guiding rational design choices. Here, we characterize gold shell-isolated nanoparticles (AuSHINs) and systematically evaluate their impact on key neutrophil end points—uptake, viability, and cell-death routing, migration, bacterial clearance assessed by an indirect regrowth assay, and NET formation—across a defined concentration range (6.4–320 μg/mL). By integrating physicochemical descriptors with functional outcomes, this study maps exposure windows in which AuSHINs alter neutrophil antimicrobial programs and provides a framework for assessing gold–silica nanoplatforms in terms of immune function alongside conventional cytotoxicity benchmarks.

## Experimental Section

### Preparation and Characterization of Nanoparticles

Gold-shell isolated nanoparticles (AuSHINs) were synthesized according to previously established protocols, with minor modifications.^26,27^ Gold nanoparticles (AuNPs) were first prepared by citrate reduction. A silica shell was subsequently formed by the addition of 3-aminopropyltrimethoxysilane (APTMS; 97%; Sigma-Aldrich; ref 281778; 1 × 10^–3^ mol/L) and sodium silicate (0.54%; Sigma-Aldrich; ref 338443) at pH 10 under continuous stirring at 90–95 °C for 3 h. To evaluate the neutrophil’s uptake of AuSHINs, they were conjugated with the photosensitizer Methylene Violet 3RAX (MV3RAX; 3-amino-7-(diethylamino)-5-phenylphenazinium chloride; 90%; Sigma-Aldrich; ref 307505) following the methodology described by Grandi et al.^28^ Because AuSHINs do not exhibit an intrinsic fluorescence signal, MV3RAX was used as a fluorescent tracer to enable intracellular tracking of the nanoparticles via MV fluorescence. After conjugation, a second silica shell was deposited using the same procedure to yield AuSHINs@MV@SiO_2_. The nanoparticles AuNPs, AuSHINs, and AuSHINs@MV@SiO_2_ were characterized by UV–vis absorption spectroscopy (CARY 60, Agilent Technologies), dynamic light scattering (DLS), and zeta potential (Zetasizer Pro, Malvern Panalytical). For measurements in biological medium, AuSHINs were dispersed (1:1, v/v) in serum-free DMEM/F12 without additional protein supplementation and analyzed at 25 and 37 °C. Unless otherwise stated, all biological and functional assays were performed using unlabeled AuSHINs. AuSHINs@MV@SiO_2_ were used exclusively for confocal microscopy experiments to enable nanoparticle tracking by fluorescence imaging.

### Isolation of Human Neutrophils

All procedures involving human blood were approved by the local Research Ethics Committee of the Faculty of Science and Letters of Assis (Approval Number: CEP-83589524.4.0000.5401). Written informed consent was obtained from all participants prior to sample collection. Human neutrophils were isolated from peripheral blood collected from self-declared healthy male donors in sodium heparin-containing tubes. Cell separation was performed using discontinuous density gradient centrifugation with Histopaque 1077 and 1119 (Sigma-Aldrich). After centrifugation, the neutrophil-enriched pellet was collected and resuspended in DMEM/F12 medium supplemented with penicillin, streptomycin and amphotericin B without fetal bovine serum (FBS). Total cell counts were determined using a Neubauer Improved Bright-Line hemocytometer (Loptik Labor).

### Nanoparticle Uptake by Neutrophils

Neutrophils (6 × 10^5^ cells) were seeded onto untreated glass coverslips in 24-well plates and incubated for 1 h at 37 °C in a humidified 5% CO_2_ atmosphere. Cells were stained with Alexa Fluor 488-conjugated wheat germ agglutinin (WGA-AlexaFluor 488; 1:2000 in PBS; Invitrogen) for 15 min, followed by two washes with PBS. AuSHINs@MV@SiO_2_ (6.4 μg/mL) were then added and incubated for 5 min. Excess noninternalized nanoparticles were removed by washing with PBS. Neutrophils were mounted using ProLong Gold Antifade Mountant with DAPI (Thermo Fisher Scientific). The cell control was not incubated with AuSHINs@MV@SiO_2_, only labeled with the markers. Imaging was performed using a STEDYCON nanoscope (Abberior Instruments). WGA-AlexaFluor 488 was used to label the plasma membrane (Ex/Em: 488/500–550 nm), DAPI was used for nuclear staining (Ex/Em: 405/417–477 nm), and AuSHINs@MV@SiO_2_ localization was assessed using a red emission channel (Ex/Em: 561/616 nm).

### Neutrophil Viability and Cell Death by Annexin V/PI Staining

Neutrophil viability and cell death following exposure to AuSHINs were evaluated by flow cytometry using Annexin V-FITC (3 μL; Invitrogen) and propidium iodide (PI) (2 μL; Invitrogen) staining. Neutrophils (6 × 10^5^ cells) were incubated with increasing concentrations of AuSHINs (6.4–320 μg/mL) for 3 h at 37 °C in a humidified 5% CO_2_ atmosphere. After incubation, cells were centrifuged for 5 min at 4 °C and resuspended in Annexin V binding buffer. Untreated cells did not receive AuSHINs and were established as cellular control. Samples were stained using Annexin V-FITC and PI and incubated for 15 min at room temperature in the dark. Flow cytometric analysis was performed using an Accuri C6 Plus flow cytometer (BD Biosciences). Cell populations were classified as follows: Annexin V-positive/PI-negative (early apoptosis), Annexin V-negative/PI-positive (necrosis), double-positive (late apoptosis), and double-negative (viable cells).

### Neutrophil Chemotaxis Assay

Neutrophil chemotaxis was assessed using a modified Boyden chamber assay, as previously described.^29,30^ Migration experiments were performed in a 48-well microchemotaxis chamber (NeuroProbe Inc.) fitted with a polycarbonate membrane (5 μm pore size). The upper wells were loaded with neutrophil suspensions (1 × 10^6^ cells/mL in DMEM/F12 medium), in the presence or absence of AuSHINs at the indicated concentrations and/or *N*-formyl-methionyl-leucyl-phenylalanine (fMLP; 10^–7^ M; Sigma-Aldrich). The lower wells contained DMEM/F12 medium alone (negative control), fMLP (positive control), AuSHINs, or AuSHINs combined with fMLP, according to the experimental condition. The chamber was incubated for 40 min at 37 °C in a humidified 5% CO_2_ atmosphere. After incubation, the membrane was removed, stained using a Panótico Rápido kit (Laborclin), and mounted on glass slides. Neutrophils that migrated to the lower surface of the membrane were quantified using ImageJ software (NIH).

### Indirect Bacterial Clearance Assay

Neutrophil phagocytic activity was evaluated using a modified protocol adapted from Green et al.^31^ *Lactobacillus crispatus* was used as the target microorganism. Neutrophils were incubated with serum-opsonized *L. crispatus* for 30 min at 37 °C in the presence or absence of AuSHINs. Bacterial survival in the supernatants was assessed after 24 h by regrowth in MRS medium followed by absorbance measurement at 560 nm using a Multiskan FC (Thermo Scientific).

### NETs Induction and Quantification

Neutrophil extracellular trap (NET) release was quantified using the PicoGreen dsDNA Assay Kit (Invitrogen) and a modified DNA–myeloperoxidase (MPO) ELISA, as previously described by da Silva Pinto et al.^32^ NETs induction was evaluated in neutrophils (5 × 10^5^ cells) incubated with increasing concentrations of AuSHINs (6.4–320 μg/mL) in serum-free DMEM/F12, in the presence or absence of phorbol 12-myristate 13-acetate (PMA; 25 nM; Sigma-Aldrich), for 3 h at 37 °C. Following incubation, supernatants were collected for quantification of NET-associated markers.

### Statistical Analysis

Experiments were performed using neutrophils isolated from independent donors. The number of biological and technical replicates for each experiment is indicated in the corresponding figure legends. Statistical analyses were performed using GraphPad Prism 8 software. Data were analyzed using one-way or two-way analysis of variance (ANOVA), followed by Bonferroni’s post hoc test, or by the nonparametric Kruskal–Wallis test followed by Dunn’s multiple comparisons test, as appropriate. Statistical significance was considered at *p* < 0.05.

## Results and Discussion

### Characterization of Gold Shell-Isolated Nanoparticles

AuSHINs were designed to combine the plasmonic properties of citrate-stabilized AuNPs with the increased colloidal stability provided by silica coating.^17,27^ Citrate reduction was selected due to its reproducibility and ability to generate negatively charged AuNPs with controlled size, suitable for subsequent silica deposition and biological applications.^7^ In addition to enabling electrostatic stabilization, citrate-capped AuNPs provide a homogeneous surface for silica growth, favoring the formation of stable core–shell nanostructures.^33^ Silica coating was employed to improve nanoparticle stability in aqueous and biological media while providing a surface for further MV conjugation. Furthermore, silica shells are known to reduce nanoparticle aggregation and preserve plasmonic properties by minimizing direct interactions between the metallic core and the surrounding environment.^33^ In addition, conjugation with Methylene Violet 3RAX (MV3RAX) enabled tracking of nanoparticles during neutrophil uptake assays.

UV–Vis extinction spectra of AuNPs, AuSHINs, and AuSHINs@MV@SiO_2_ recorded in ultrapure water are shown in Figure 1A. Bare AuNPs displayed a characteristic localized surface plasmon resonance (LSPR) band centered at 520 nm. Following silica coating, the LSPR maximum red-shifted to 526 nm, consistent with changes in the local refractive index surrounding the gold core.^34^ Such plasmonic shifts are commonly observed after dielectric coating and reflect modifications in the optical environment surrounding the metallic nanoparticle surface.^35^ After photosensitizer conjugation and deposition of an additional silica shell, a further red shift to 528 nm was observed for AuSHINs@MV@SiO_2_. The small magnitude of this additional shift indicates that the plasmonic core remained structurally preserved and is consistent with an increased effective separation between the gold surface and the outer dielectric environment, over which the plasmonic electromagnetic near-field decays exponentially.^36^ Dynamic light scattering (DLS) (Figure 1B) revealed a progressive increase in hydrodynamic diameter from 21.44 ± 0.20 nm (AuNPs, PDI: 0.14) to 28.56 ± 1.53 nm (AuSHINs, PDI: 0.22) and 46.67 ± 2.53 nm (AuSHINs@MV@SiO_2_, PDI: 0.31), confirming the stepwise assembly of the hybrid nanoparticles. The moderate increase in PDI values after each step remained compatible with colloidally nanosystems, indicating that the selected coating conditions preserved nanoparticle dispersion while enabling progressive surface modification.^33^ The substantial increase observed after the second coating step further supports photosensitizer conjugation and silica shell formation. Zeta potential measurements indicated negatively charged surfaces for all nanoparticles, with values of −43.08 ± 2.17 mV (AuNPs), −46.72 ± 1.82 mV (AuSHINs), and −46.02 ± 0.01 mV (AuSHINs@MV@SiO_2_). The slight shift toward more negative potential after silica deposition is consistent with surface silanol groups and suggests enhanced colloidal stability in aqueous media.^33^ The preservation of strongly negative surface charge throughout the synthesis and conjugation steps indicates that the selected preparation strategy maintained electrostatic stabilization while allowing successful surface engineering of the nanostructures.^33^

**Figure 1:**
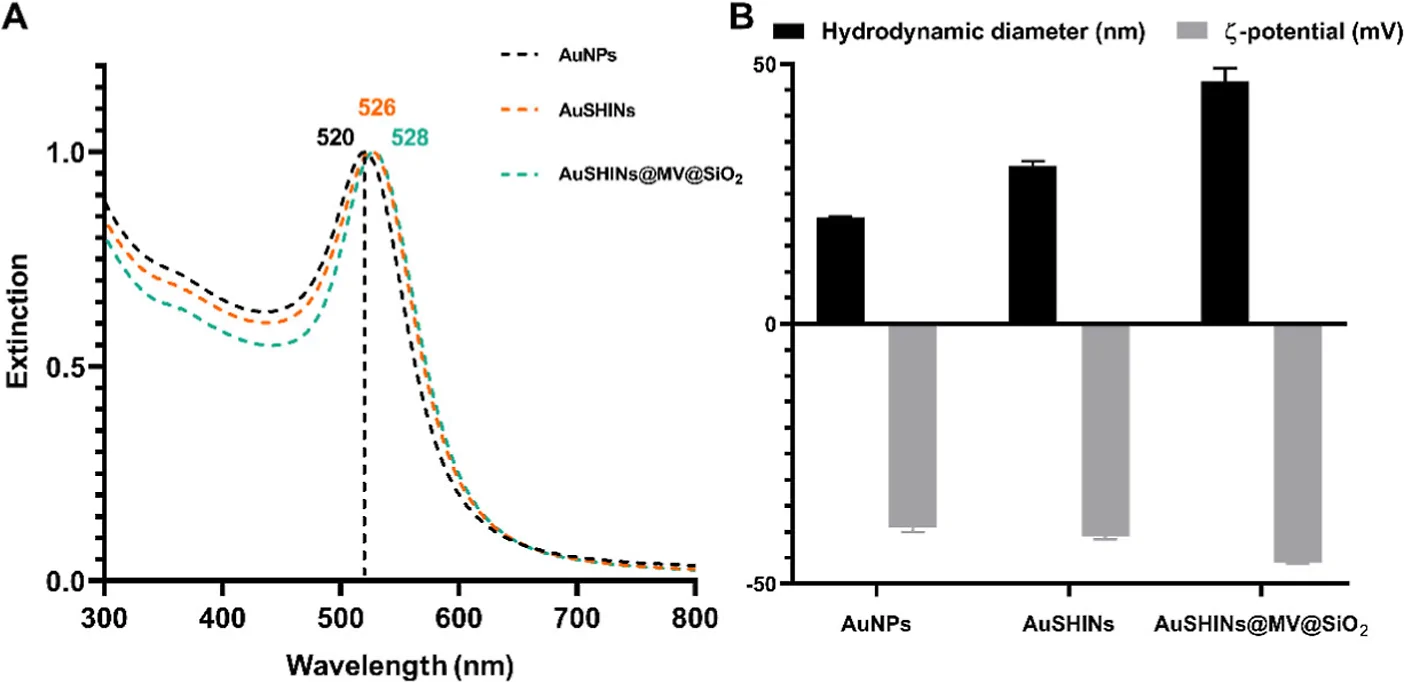
Physicochemical characterization of AuNPs, AuSHINs, and AuSHINs@MV@SiO_2_. (A) UV–vis extinction spectra of AuNPs, AuSHINs and AuSHINs@MV@SiO_2_ in ultrapure water showing the progressive plasmonic red-shift following silica coating and photosensitizer incorporation (B) Hydrodynamic diameter and zeta potential values in ultrapure water (1:1, v/v) at 25 °C. Values represent mean ± SD of two independent analyses, each measured in triplicate.

To further evaluate nanoparticle’s behavior under biologically relevant conditions, additional DLS and zeta-potential analyses were performed in ultrapure water and serum-free DMEM, both at 25 and 37 °C (Table 1). In ultrapure water, AuSHINs exhibited hydrodynamic diameters of 27.25 ± 0.13 nm and 29.00 ± 0.65 at 25 and 37 °C, respectively, with corresponding zeta-potential values of −40.15 ± 1.69 mV and −37.47 ± 3.09. When dispersed in DMEM, the nanoparticles had diameters of 38.04 ± 1.20 nm at 25 °C and 33.05 ± 5.05 nm at 37 °C, accompanied by less negative zeta-potential values of −21.55 ± 0.55 mV and −23.37 ± 0.75 mV, respectively. Because this formulation contained neither serum nor other protein supplements, the observed changes are more likely associated with ionic screening and interactions with nonprotein medium constituents than with protein-corona formation. Similar reductions in zeta potential and increases in hydrodynamic diameter in biologically relevant media have been described for gold-based nanomaterials and are commonly associated with the establishment of dynamic nanobio interfaces, including ionic screening effects, adsorption of medium components, changes in colloidal organization, and, depending on medium composition, protein adsorption.^37,38^ Therefore, despite these slight alterations, AuSHINs maintain colloidal stability in different media. Moreover, only modest variations were observed between 25 and 37 °C, indicating limited temperature-dependent effects on nanoparticle stability within the evaluated range.

**Table 1:** Hydrodynamic Diameter and Zeta Potential of AuSHINs Dispersed (1:1, v/v) in Ultrapure Water and in Serum- and Protein-Free DMEM/F12 at 25 and 37 °C.

	Ultrapure water		DMEM	
	25 °C	37 °C	25 °C	37 °C
Hydrodynamic diameter (nm)	27.25 ± 0.13	29.00 ± 0.65	38.04 ± 1.20	33.05 ± 5.05
Zeta potential (mV)	–40.15 ± 1.69	–37.47 ± 3.09	–21.55 ± 0.55	–23.37 ± 0.75

### Cellular Internalization of AuSHINs by Neutrophils

Determining whether AuSHINs interact with neutrophils is essential for interpreting their potential immunomodulatory effects. As professional phagocytes, neutrophils are capable of recognizing and internalizing nanoparticles through multiple mechanisms, including phagocytosis, macropinocytosis, and clathrin-independent pathways.^21,39^ To assess cellular uptake, freshly isolated human neutrophils were incubated with AuSHINs@MV@SiO_2_ at 6.4 μg/mL for 5 min and then analyzed by confocal fluorescence microscopy (Figure 2). The plasma membrane and nuclei were labeled with Alexa Fluor 488-conjugated wheat germ agglutinin and DAPI, respectively. A distinct red fluorescence signal corresponding to AuSHINs@MV@SiO_2_ was observed within the cytoplasm and in the perinuclear region, supporting rapid nanoparticle association with and/or internalization by neutrophils following cell contact. Nanoparticle uptake by neutrophils is known to depend strongly on particle size and surface coating.^40–42^ Because AuSHINs@MV@SiO_2_ contain MV3RAX and an additional silica layer, their uptake behavior may differ from that of the unlabeled AuSHINs used in the functional assays. Therefore, the confocal data provide qualitative evidence of rapid association/internalization of the fluorescent formulation but do not establish equivalent uptake kinetics or internalization mechanisms for unlabeled AuSHINs. Previous studies have shown that AuSHINs (1 × 10^12^ NPs mL^–1^; 64 μg mL^–1^) internalized by breast cancer cell lines (MDA-MB-231 and MCF-7) were trafficked to lysosomal compartments within 2 h of incubation, indicating processing through the endolysosomal pathway.^37^ This intracellular routing is characteristic of phagocytic or endocytic mechanisms and suggests that similar vesicular trafficking may occur in neutrophils. However, the present data, limited to a 5 min observation window, do not establish sustained intracellular localization or endolysosomal stability and cannot exclude premature lytic swelling. These questions will require longer-term investigation.

**Figure 2:**
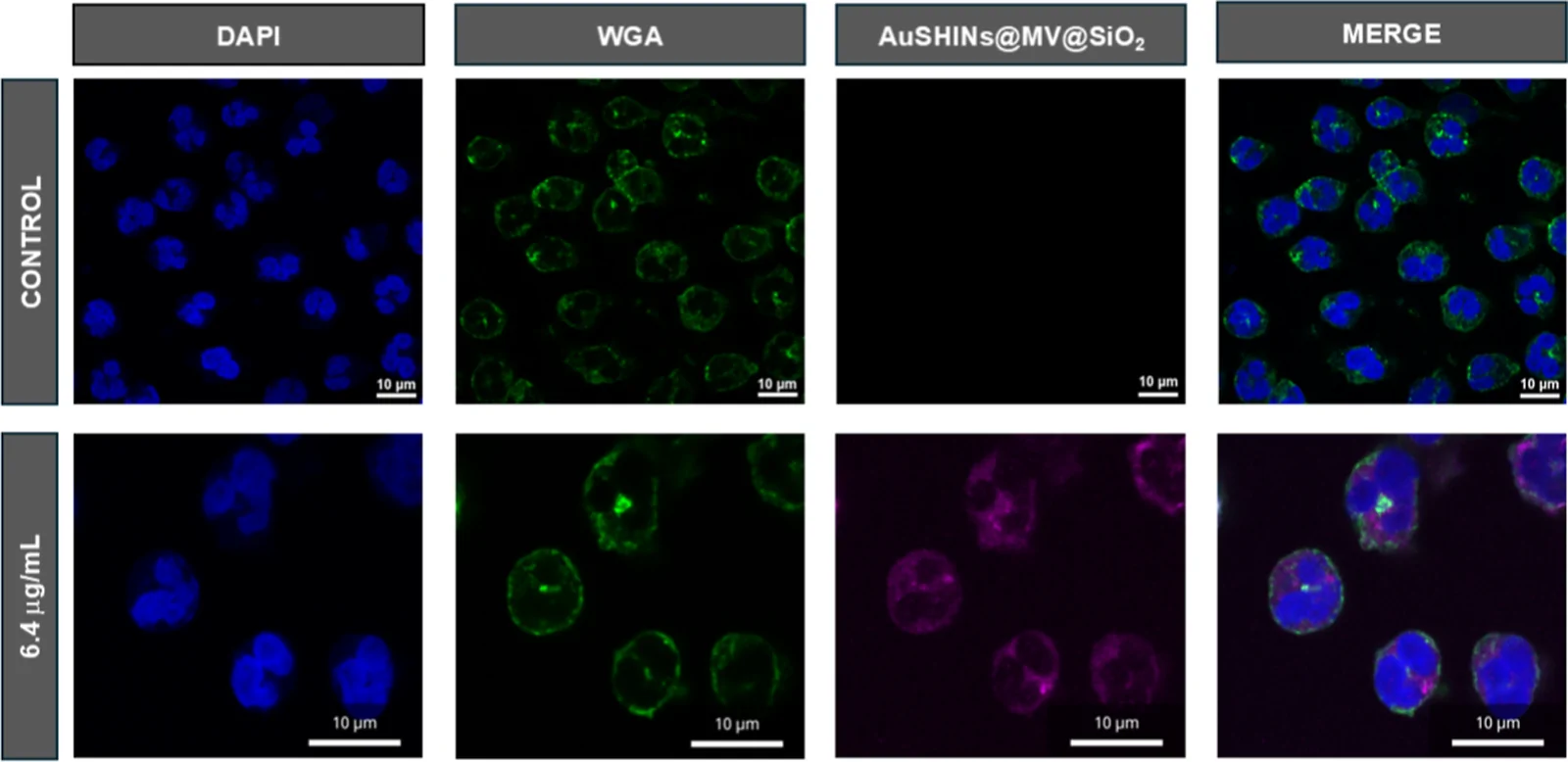
Confocal fluorescence microscopy of AuSHINs internalization by neutrophils. Neutrophils adhered to glass coverslips were incubated with AuSHINs@MV@SiO_2_ (6.4 μg/mL) for 5 min at 37 °C to evaluate nanoparticle uptake. Nuclei are shown in blue (DAPI), plasma membranes in green (WGA–Alexa Fluor 488), and AuSHINs@MV@SiO_2_ in purple through MV fluorescence. Representative images demonstrate the rapid association and/or internalization of nanoparticles by neutrophils.

### Impact of AuSHINs on Neutrophil Viability

Freshly isolated neutrophils (6 × 10^5^ cells) were incubated for 3 h with increasing concentrations of AuSHINs (6.4–320 μg/mL). Trypan blue exclusion revealed that viability remained comparable to untreated controls across most concentrations of AuSHINs, with a modest but statistically significant reduction observed only at 320 μg/mL (15.91% decrease; Figure 3A). This profile is consistent with previous reports indicating that silica coating improves the colloidal stability and overall biocompatibility of gold nanoparticles.^42,43^ To determine the predominant cell-death pathway, Annexin V/propidium iodide staining was performed followed by flow cytometry analysis (Figure 3B–D). AuSHINs induced a dose-dependent increase in early apoptotic (Annexin V^+^/PI^–^) neutrophils, reaching statistical significance at concentrations ≥32 μg/mL. Specifically, exposure to 32, 64, and 320 μg/mL resulted in 37.15%, 59.01%, and 63.08% early apoptotic neutrophils, respectively. Late apoptotic populations (Annexin V^+^/PI^+^) were significantly elevated at 64 and 320 μg/mL (5.06% and 26.05%, respectively), whereas necrotic cells (Annexin V^–^/PI^+^) remained unchanged across all tested conditions. A comparable pro-apoptotic response has been reported for AuSHINs (1 × 10^12^ NPs/mL, 64 μg/mL) exposure in breast cancer cell lines (MDA-MB-231 and MCF-7 cells), where nanoparticle treatment increased early apoptosis in a time-dependent manner, with minimal induction of necrosis.^37^ These cross-cell-type observations suggest that gold-silica nanoparticles preferentially activate regulated apoptotic pathways rather than inducing nonspecific membrane damage.

**Figure 3:**
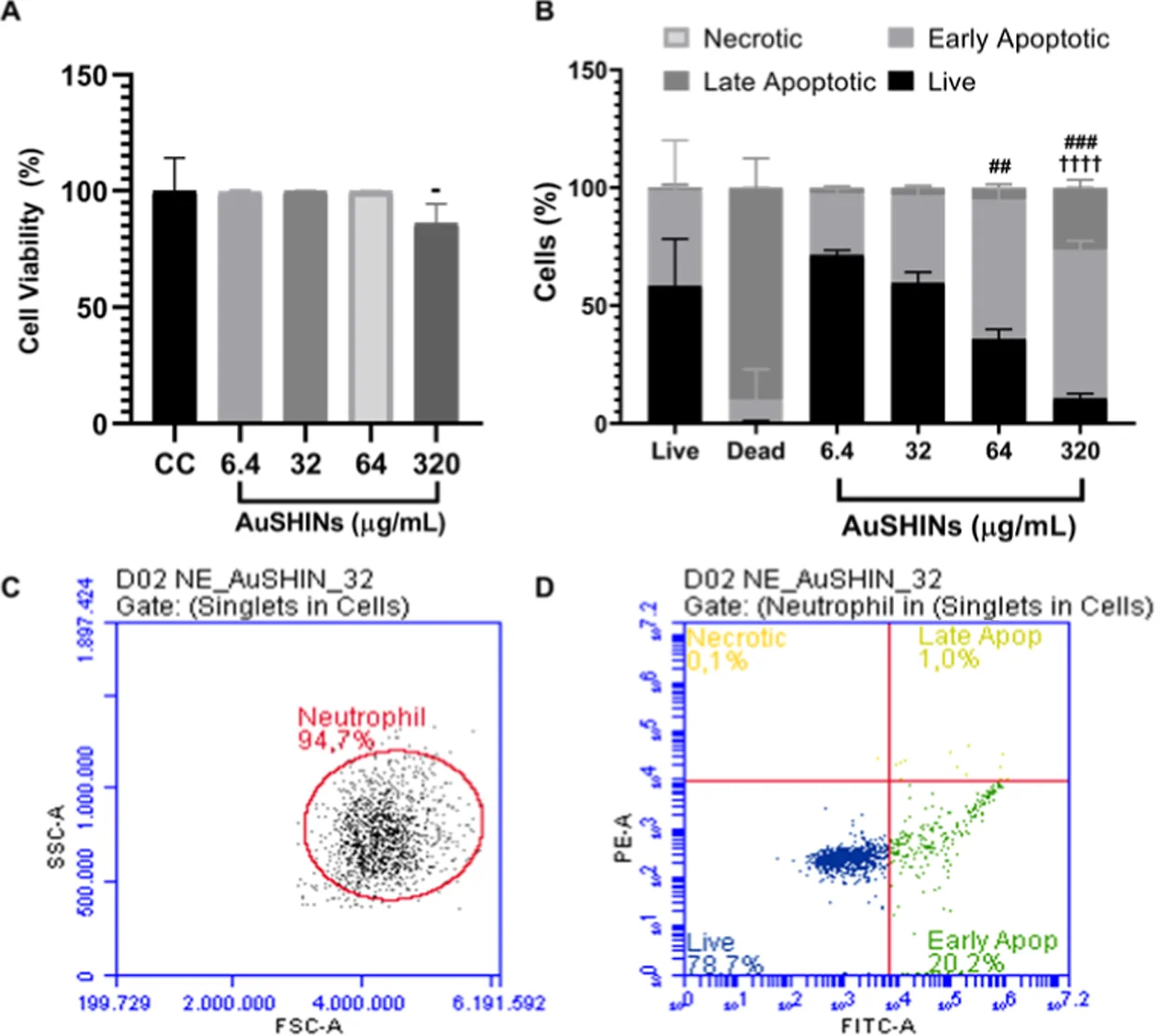
Effects of AuSHINs on neutrophil viability. (A) Neutrophil viability determined by Trypan blue exclusion assay after 3 h of incubation with increasing concentrations of AuSHINs. Data are presented as mean ± SD from four independent donors (*n* = 4), each analyzed in technical duplicates. Statistical analysis was performed using the Kruskal–Wallis test followed by Dunn’s multiple comparisons test. Comparisons were performed against the untreated cell control (CC). * indicates *p* = 0.0317 relative to CC. (B) Neutrophil viability and cell death assessed by Annexin V/PI flow cytometry after 3 h of incubation with AuSHINs. Data are presented as mean ± SD from two independent donors (*n* = 2), each analyzed in technical duplicate. Statistical analysis was performed using two-way ANOVA followed by Bonferroni’s multiple comparisons test. Each cell population (live, early apoptotic, late apoptotic, and necrotic cells) from each treatment group was compared with the corresponding cell population of the untreated control group (Live). Symbols indicate significant differences relative to the respective untreated population: ##, *p* = 0.0028; ###, *p* = 0.0002; ††††, *p* < 0.0001. ″#″ denotes increased early apoptotic cells and ″†″ denotes increased late apoptotic cells. (C,D) Representative flow cytometry gating strategy and quadrant analysis used to identify viable, early apoptotic, late apoptotic, and necrotic neutrophils.

From an immunological standpoint, apoptosis is a regulated mechanism for neutrophil clearance and contributes to the resolution of inflammation, since apoptotic neutrophils are efficiently engulfed by macrophages and can promote the release of anti-inflammatory mediators such as TGF-β and IL-10.^44^ Apoptosis has also been reported in human neutrophils after exposure to gold nanoparticles.^45,46^ In contrast, necrosis is typically linked to membrane rupture and amplification of inflammatory responses.^47,48^ The lack of a necrotic population in our conditions therefore suggests that AuSHINs preferentially engage controlled apoptotic programs rather than lytic cell death. Overall, AuSHINs showed good biocompatibility at low-to-moderate concentrations and induced dose-dependent apoptotic signaling at higher concentrations, without evidence of necrotic cytotoxicity, which may be relevant when interpreting their effects on neutrophil inflammatory functions.

### AuSHINs-Induced Neutrophil Migration

Having established that AuSHINs were internalized without compromising viability at subcytotoxic concentrations, we next investigated whether these nanostructures modulate key neutrophil effector functions. Neutrophil migration was assessed using a Boyden chamber assay under different configurations (Figure 4A). When AuSHINs were placed in the lower chamber, all tested concentrations (6.4–320 μg/mL) significantly promoted neutrophil migration compared to medium controls, reaching levels comparable to the classical chemoattractant fMLP (Figure 4B). These results indicate that AuSHINs promote neutrophil transmigration across the Boyden chamber and are consistent with previous reports describing properties of gold-based nanoparticles.^49,50^

**Figure 4:**
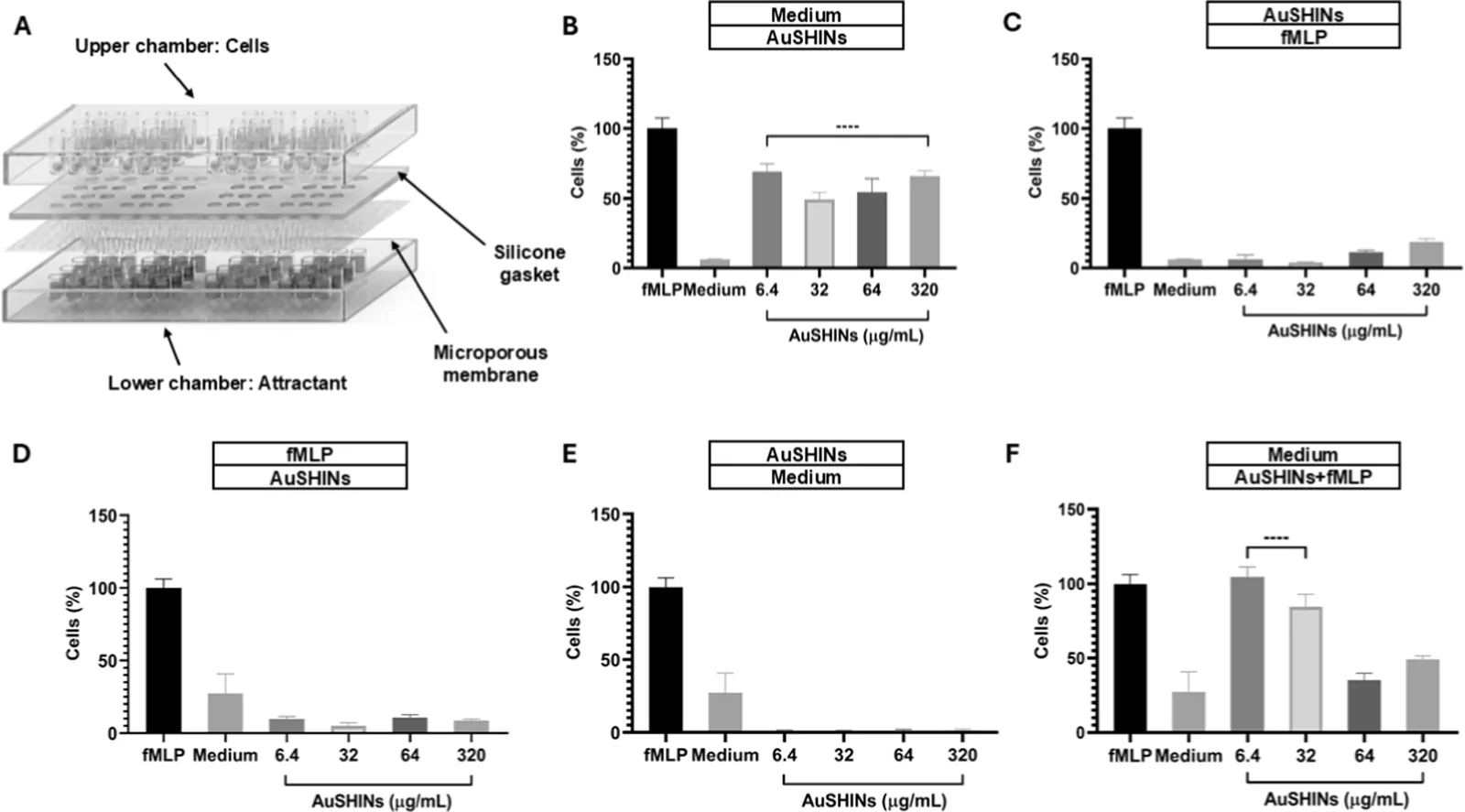
Chemotactic profile of AuSHINs in neutrophils. (A) Schematic representation of the Boyden chamber assay. (B) Migration of neutrophils toward AuSHINs. (C) Migration of AuSHINs-treated neutrophils toward fMLP. (D) Migration of fMLP-stimulated neutrophils toward AuSHINs. (E) Migration of AuSHINs-treated toward medium alone. (F) Migration of neutrophils toward a combination of AuSHINs and fMLP. Data are presented as mean ± SD from two independent donors (*n* = 2), each analyzed in technical triplicate. Statistical analysis was performed using one-way ANOVA followed by Bonferroni’s multiple comparisons test. In panels B–F, each experimental condition was compared with the corresponding medium control group for the respective assay. Significant increases in migration relative to medium are indicated by “----” (*p* < 0.0001).

In contrast, neutrophil migration was markedly reduced when AuSHINs and fMLP were placed in opposing compartments. Migration was inhibited both when AuSHINs-treated neutrophils were exposed to an fMLP gradient (Figure 4C) and when fMLP-activated neutrophils encountered AuSHINs in the lower chamber (Figure 4D). Neutrophils pretreated with AuSHINs also failed to migrate under null gradient conditions (Figure 4E). These findings suggest that AuSHIN-induced effects on neutrophil migration are dependent on the experimental configuration. One possible explanation is that, when AuSHINs and fMLP are distributed in opposing compartments, neutrophils are exposed to competing migratory cues, resulting in reduced transmigration across the Boyden chamber. This interpretation is compatible with the well-established hierarchy of neutrophil chemoattractants, in which bacterial-derived formyl peptides such as fMLP act as high-priority migratory signals.^51,52^ However, because Boyden chamber assays do not discriminate between directional chemotaxis and altered nondirectional motility, these observations should be interpreted cautiously.^53^ Consistent with these interpretation, previous studies have shown that gold and other metallic nanoparticles, such as AuNP-IgG and AgNPs, can inhibit neutrophil migration induced by inflammatory stimuli.^43,54^ Notably, when both AuSHINs and fMLP were present in the lower compartment, neutrophil migration remained elevated (Figure 4F). This observation suggests that the reduced transmigration observed in the opposing-compartment configurations cannot be attributed solely to a generalized loss of migratory capacity.

### AuSHINs Differently Affected Neutrophil Microbicidal Functions

Upon reaching inflammatory sites, neutrophils deploy multiple antimicrobial strategies, including phagocytosis and the release of neutrophil extracellular traps (NETs). To assess whether AuSHINs affect neutrophil-mediated bacterial clearance, neutrophils were incubated with serum-opsonized *L. crispatus* in the presence or absence of AuSHINs (Figure 5). Bacterial recovery after neutrophil incubation was quantified by regrowth in MRS medium, an approach that provides a functional measure of bacterial clearance under physiologically relevant opsonization conditions. Untreated neutrophils reduced bacterial recovery by approximately 50% relative to bacterial control (Figure 5A). In contrast, neutrophils exposed to AuSHINs displayed bacterial levels comparable to bacterial-only controls across all tested concentrations, indicating a marked reduction in neutrophil-mediated bacterial clearance. While this indirect assay does not discriminate between impaired bacterial uptake, defective intracellular killing, or alterations in postphagocytic processing, the magnitude of bacterial persistence suggests that AuSHINs interfere with early neutrophil antimicrobial responses associated with bacterial clearance. To our knowledge, the effect of AuSHINs on neutrophil-mediated phagocytosis has not yet been directly addressed. Previous reports have described preserved phagocytic activity in neutrophils exposed to gold nanoparticles.^46^ The combination of gold with graphene oxide significantly increased the phagocytosis of *Staphylococcus aureus* and *Escherichia coli*, elevating the clearance of both types of bacteria by phagocytic immune cells.^55^

**Figure 5:**
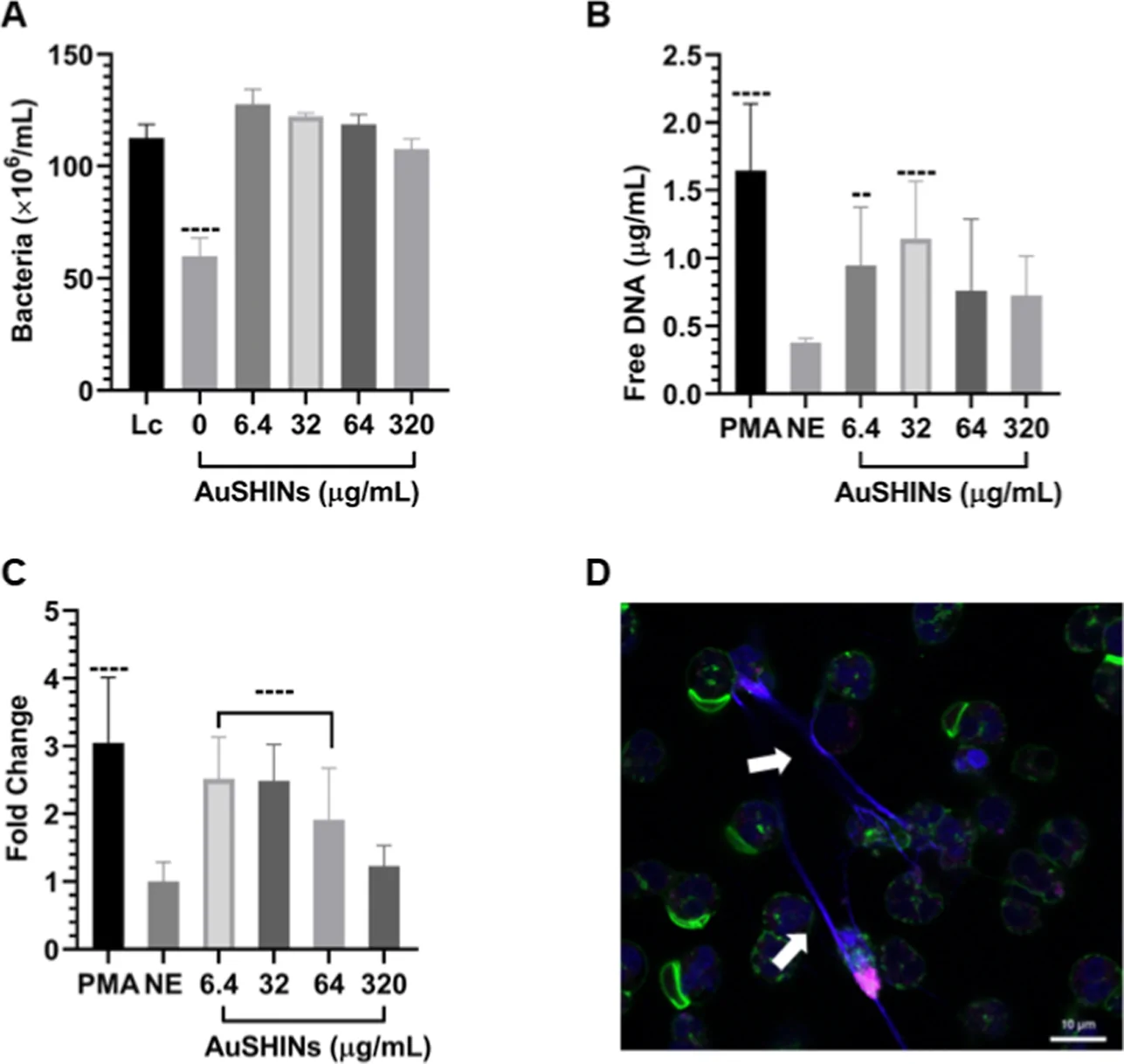
Interference of neutrophil microbicidal functions by AuSHINs. (A) Indirect bacterial clearance assay using *Lactobacillus crispatus* as target bacteria. Bacterial survival was determined by regrowth in MRS medium after 24 h and quantified by absorbance at 560 nm. Data are presented as mean ± SD from two independent donors (*n* = 2), each analyzed in technical duplicate. Statistical comparisons were performed relative to the bacterial control group (*L. crispatus* in the absence of neutrophils; Lc), and reductions in bacterial growth are indicated by “----” (*p* < 0.0001). (B) Quantification of extracellular DNA released by neutrophils following stimulation with AuSHINs, measured using the PicoGreen dsDNA assay. Data are presented as mean ± SD from five independent donors (*n* = 5), each analyzed in technical triplicate. Statistical analysis was performed using the Kruskal–Wallis test followed by Dunn’s multiple comparisons test. Comparisons were performed relative to the spontaneous NET release control group (NE). Significant increases in extracellular DNA release are indicated by “----” (*p* < 0.0001) and “--” (*p* = 0.0048). (C) Quantification of NET-associated DNA–myeloperoxidase (DNA–MPO) complexes by modified MPO ELISA. Data are presented as mean ± SD from five independent donors (*n* = 5), each analyzed in technical triplicate. Statistical analysis was performed using one-way ANOVA followed by Bonferroni’s multiple comparisons test. Comparisons were performed relative to the spontaneous NET release control group (NE). Significant increases in DNA–MPO complex formation are indicated by “----” (*p* < 0.0001). (D) Representative confocal microscopy image showing AuSHIN-induced NET release. Extracellular DNA is shown in blue, AuSHINs in red, and cell membranes in green. NET-like structures are indicated by white arrows. Scale bar = 10 μm.

We next investigated whether AuSHINs influence NET formation. Neutrophils were exposed to increasing concentrations of AuSHINs (6.4–320 μg/mL) or PMA as a positive control, and NET release was quantified using extracellular DNA measurements and ELISA-based detection of DNA–MPO complexes (Figure 5B,C). At 6.4 and 32 μg/mL, AuSHINs significantly enhanced NET formation, reaching levels comparable to PMA stimulation. A representative image illustrating NET structures formed under these conditions is shown in Figure 5D. In contrast, higher concentrations failed to promote NET release above baseline. The increase in NET formation occurred in parallel with impaired phagocytic bacterial clearance, suggesting a shift in neutrophil antimicrobial output rather than a global loss of cellular competence.^56,57^ This shift from intracellular phagocytic clearance toward extracellular trap–mediated responses may have important implications for microbial containment and inflammatory outcomes, depending on the biological context.^58^ NETs are capable of capturing and/or inactivating microorganisms.^18^ This response may partially compensate for impaired phagocytic clearance, although this possibility requires direct functional validation.

Importantly, at 64 and 320 μg/mL, the reduction in NET release coincided with increased apoptotic signaling, particularly at the highest concentration. This association suggests that the attenuation of neutrophil effector responses at higher exposure levels may reflect cellular stress rather than direct inhibition of the NETosis machinery. Overall, the distinct effects observed on cell viability, bacterial clearance, and NET formation indicate that AuSHINs do not uniformly activate or suppress neutrophil responses, emphasizing the importance of interpreting each outcome within its respective exposure range.

## Conclusion

Gold shell-isolated nanoparticles (AuSHINs) produced concentration-dependent changes in human neutrophil function under the experimental conditions evaluated. The nanostructures exhibited hydrodynamic diameters ranging from 28 to 46 nm, negative surface charge, and colloidal stability, and showed rapid association with and/or internalization by neutrophils. At subcytotoxic concentrations, AuSHINs preserved cell viability while reshaping antimicrobial activity, reducing phagocytic bacterial clearance and enhancing NET release, consistent with a shift toward extracellular defense mechanisms. In contrast, higher concentrations promoted apoptotic signaling without necrosis, thereby limiting inflammatory lysis while attenuating effector functions. These findings indicate that the impact of AuSHINs on neutrophils extends beyond cytotoxicity alone, with differential effects on antimicrobial-related functions observed across concentration windows. Collectively, these findings highlight the importance of considering nanoparticle physicochemical properties when evaluating neutrophil responses to gold-based nanomaterials. Defining these concentration windows and functional outcomes is essential for the design of gold-based nanoplatforms intended for immunomodulatory applications, where controlled interaction with innate immune cells is critical for therapeutic efficacy and safety.

## Data Availability

The data supporting the findings of this study are publicly available in the Zenodo repository at 10.5281/zenodo.22255593. The data set includes the individual experimental data underlying the figures presented in this study, as well as the original GraphPad Prism files used for data analysis and figure generation.
